# Constrictive Pericarditis–A Cloak Camouflaging Lymphoma

**DOI:** 10.3390/hematolrep15010017

**Published:** 2023-03-02

**Authors:** Delanthabettu Venugopala, Nikhil Victor Dsouza, Vishak Acharya, Maneesh Rai, Chaithra Gowthuvalli Venkataramana, Stergios Boussios

**Affiliations:** 1 Department of Internal Medicine, Kasturba Medical College, Manipal Academy of Higher Education, Manipal, Mangalore 576104, India; 2 Department of Respiratory Medicine, Medway NHS Foundation Trust, Windmill Road, Gillingham ME7 5NY, UK; 3 Department of Pulmonary Medicine, Kasturba Medical College, Manipal Academy of Higher Education, Manipal, Mangalore 576104, India; 4 Department of Cardiology, Kasturba Medical College, Manipal Academy of Higher Education, Manipal, Mangalore 576104, India; 5 Department of Pathology, Kasturba Medical College, Manipal Academy of Higher Education, Manipal, Mangalore 576104, India; 6 Department of Medical Oncology, Medway NHS Foundation Trust, Windmill Road, Gillingham ME7 5NY, UK; 7 School of Cancer & Pharmaceutical Sciences, Faculty of Life Sciences & Medicine, King’s College London, London SE1 9RT, UK; 8 Kent Medway Medical School, University of Kent, Canterbury CT2 7LX, UK; 9 AELIA Organization, 9th Km Thessaloniki–Thermi, 57001 Thessaloniki, Greece

**Keywords:** constrictive pericarditis, diffuse large B cell lymphoma, FDG PET-CT scan, diagnostic challenge, primary cardiac lymphoma

## Abstract

Non-Hodgkin’s lymphoma presenting as a primary cardiac lymphoma (PCL) is extremely unusual. Having a predilection for the right side of the heart and accounting for 1% of all cardiac tumours, the difficulty in diagnosing the lesion, owing to the location and vague presenting symptoms and signs, often leads to delayed diagnosis and poor prognosis. In our case report, a middle-aged male was diagnosed with PCL presenting as pyrexia of unknown origin with the help of F18-fluorodeoxyglucose positron emission tomography (18 FDG-PET). PET-CT is an invaluable tool in patients with pyrexia of unknown origin (PUO), especially caused by neoplasms as it helps in localizing the target lesion, aiding in selecting the appropriate intervention for rapid tissue diagnosis. This case serves to sensitize the physicians of PCL presenting with PUO and mimicking a relatively common cardiac tumour such as atrial myxoma.

## 1. Introduction

One of the very rare forms of non-Hodgkin’s lymphoma (NHL) is primary cardiac lymphoma (PCL). According to World Health Organization (WHO), in 2015 it was defined as a large lymphoma mass in the heart with or without secondary lesions in other areas of the body. Primary cardiac tumours being rare, with an incidence of 0.056%, only 10% of them are malignant, with PCL accounting for 1% [1].

PCL usually manifests with cardiac and mediastinal symptoms leading to a relatively early disease localisation. PCL presenting as pyrexia of unknown origin (PUO), as in our case, is extremely unusual. Early identification and diagnosis are crucial in disease prognostication as the clinical manifestations are nonspecific and location dependant. Despite the availability of modern imaging modalities and biopsy techniques, outcomes rely on histopathology and early treatment. If not treated in time, PCL is fatal [2].

## 2. Case Presentation

A 55-year-old gentleman presented with fever for one month, intermittent in nature with an evening rise. It was associated with cough and weight loss of 4 kg over 2 months. No history of addictions or drug abuse. On examination, general physical and vitals were unremarkable. Respiratory examination revealed rhonchi bilaterally with no organomegaly and normal cardiovascular assessment. Initial investigations for PUO evaluation revealed elevated erythrocyte sedimentation rate (ESR) of 96 (0–10 mm/h), C-reactive protein (CRP) of 116 (<5 mg/L) and LDH (lactate dehydrogenase) of 386 (0–250 U/L). Endemic conditions such as malaria, dengue, leptospirosis, and tuberculosis (TB) were ruled out. Further tests including human immunodeficiency virus (HIV), hepatitis B surface antigen (HbsAg), hepatitis C virus (HCV), antinuclear antibodies (ANA), procalcitonin, and blood cultures were negative.

**Figure 1:**
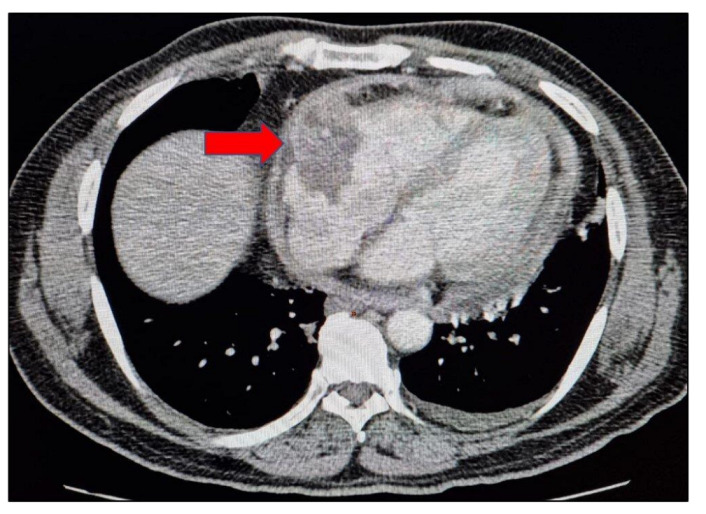
Mediastinal axial view of the thorax showing pericardial thickening and heterogeneous area protruding from anterior visceral pericardium and bulging partially into right atrium and tricuspid valve region.

Radiological imaging, namely chest X-ray and high-resolution computerised tomography (HRCT) of the chest, showed no significant findings. To rule out endobronchial TB or malignancy, a bronchoscopy was performed, which was normal. A 2D echo was conducted to rule out endocarditis but, to our surprise, it unearthed massive pericardial effusion with deposits and pericardial thickness of 9 mm. Contrast enhanced computerised tomography (CECT) of the abdomen revealed minimal left sided pleural effusion with pericardial thickening with mild enhancement and measuring 9–10 mm in thickness, sub-centimetric mesenteric, and bilateral inguinal lymph nodes. An heterogeneous area measuring about 47 × 35 mm protruding from anterior visceral pericardium and bulging partially into right atrium and tricuspid valve region was noted (Figure 1).

**Figure 2:**
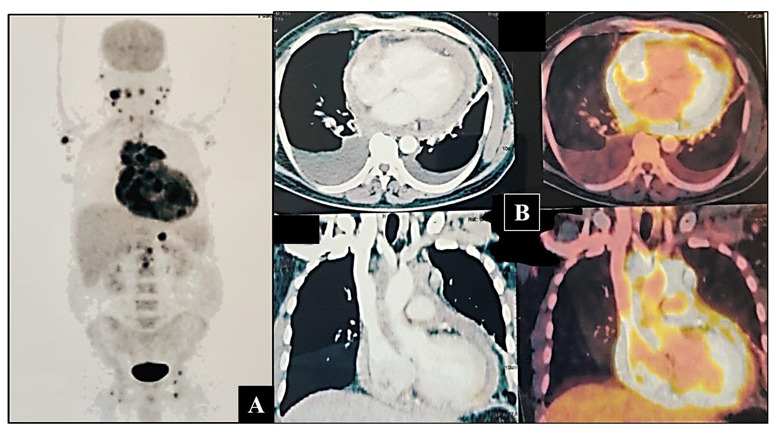
(**A**) Maximum intensity projection (MIP) image of 18FDG PET-CT scan showing increased uptake of FDG around the cardia, neck, axillary, and inguinal nodes. (**B**) Axial and coronal sections showing increased uptake by the lesion, surrounding cardia, and great vessels.

This was followed up with a contrast-enhanced F18-fluorodeoxyglucose positron emission tomography (18 FDG-PET) scan which disclosed metabolically active disease—soft tissue lesion involving the entire pericardium, left ventricular myocardium with soft tissue infiltration into the right atrium. It was extending into the superior mediastinum and encasing the ascending aorta, aortic arch, and pulmonary artery. Hyper-metabolic mediastinal, right cardio-phrenic, abdominal, bilateral inguinal, axillary, and cervical nodes were seen (Figure 2A,B).

**Figure 3:**
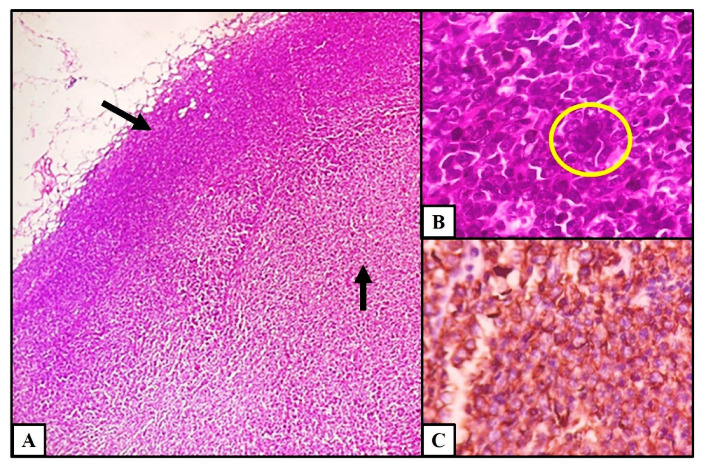
Histopathology findings of lymph node. (**A**) Effacement of lymph node architecture by atypical lymphoid cells (H&E stain, 4×). (**B**) Large RS-like cell with multilobated nucleus (H&E stain, 40×). (**C**) Expression of CD20 in atypical lymphoid cells (IHC, 20×).

Axillary lymph node biopsy and histopathology showed effaced lymph node architecture by sheets of atypical lymphoid cells (Figure 3A). Scattered large reed Sternberg (RS)-like cells with multilobated nucleus, coarse vesicular chromatin, and prominent nucleoli were seen (Figure 3B). Atypical lymphoid cells were strongly positive for CD20 on immunohistochemistry (Figure 3C) and negative for CD3, TdT, ALK, Epstein Barr virus (EBV), CD10, and BCL6 with 65% MIB index. A final diagnosis of diffuse large cell lymphoma (DLBCL) not otherwise specified activated B cell type was provided based on morphology and immunohistochemistry, Ann-Arbor stage IV, and IPI (International Prognostic Index) score of 3.

The patient was started on R-CHOP (rituximab, cyclophosphamide, hydroxydaunorubicin hydrochloride (doxorubicin hydrochloride), vincristine (Oncovin) and prednisone) chemotherapy regimen. After 2 cycles of treatment, an interim PET-CT scan was performed which revealed near total resolution of the disease with mild pericardial thickening. He is presently continuing treatment and is on regular follow up. On follow up, an ECHO was performed which showed resolution of the effusion and decrease in the number and size of deposits.

## 3. Discussion

Extranodal DLBCL arises from extranodal organs in about 40% of cases [3]. The diagnosis is challenging because the symptoms are similar to many other diseases of inflammatory aetiology [4]. Primary tumours of the cardia are extremely rare and account for less than 1% of unselected patients on autopsy. Amongst them 75% are benign and 50% being myxomas [5]. Tumours other than myxomas as diagnosed in our case are extremely uncommon. So far, there have been 47 reported cases of fibrin-associated DLBCL in the literature [6]. This is a rare EBV-related lymphoproliferative disorder, arising within fibrinous material in different clinical settings. From the diagnostic perspective, it disclosed incidentally on histological evaluation of surgical specimens removed for other diseases, given that it is not mass-forming. Cardiac myxoma represents one of the most frequent sites of occurrence. It can also arise within a pseudocyst, or in association with a prosthetic cardiac valve or a metallic implant [7]. Therapeutically, it can be considered surveillance following surgical resection of the myxoma, whilst systemic chemotherapy is recommended when there is evidence of disease recurrence [8].

Cardiac and pericardial involvement by malignant lymphoma constitutes approximately 1% of cardiac tumours and 0.5% of extra-nodal NHL, with DLBCL being the most common type [9]. Epidemiological characteristics of PCL, as per a recent systematic review accomplished in 2020, reported mean age of 62, male preponderance with higher prevalence of cases in the Asian followed by European region (48% vs. 27%) [10].

PCL is extremely rare, as described in the literature, and presents as either primary, arising from the cardia or secondary to infiltrating lymphoma from adjacent structures. The diagnostic criteria for PCL have progressively broadened over time. Initially PCL was recognised as a mass with exclusive involvement of the heart or pericardium. While presently, the definition has been redefined and expanded to include lesions when the primary bulk of the lymphoma is localised in the heart or pericardium even if limited extracardiac lymphoma is present and the clinical manifestations are related primarily to cardiac dysfunction [11,12,13].

The pathogenesis of this condition is still being researched, although certain studies have proposed the involvement of congenital immune deficiencies and infections such as HIV, EBV, etc. The clinical manifestation of PCL is nonspecific due to the fact that it depends on the location of the tumour. It mostly involves structures in the right side of the heart, namely the atrium and pericardium [13,14].

PCL as a disease has a predilection for the right heart with a likely right atrium involvement. Pericardial effusion is often present though constrictive pericarditis is rarely reported. Clinical manifestations of PCL are often non-specific, accounting for delayed diagnosis and poor prognosis. The most common symptom is dyspnoea due to compression effect or heart failure. Other clinical signs and symptoms depend largely on the localisation of the tumour (e.g., superior vena cava obstruction with right heart involvement), infiltration of the myocardium (causing arrhythmias and angina) [15]. However, the unique feature in our case is the manifestation of fever with constrictive pericarditis. Common cardiac tumours including atrial myxoma often present with PUO too and may be mistaken and difficult to differentiate from rarer entities such as cardiac lymphoma. As the diagnosis of these conditions hinges primarily on imaging, delayed diagnosis of these radiologically similar entities may be catastrophic with serious medico-legal implications [16].

Since the clinical diagnosis is difficult in PCL, the use of modern non-invasive imaging tests is necessary. Transoesophageal echocardiography (TEE), CT, and magnetic resonance imaging (MRI) are very useful in the identification of location, size, and extension of the lesion. However, FDG PET-CT has emerged as a better tool as it has the ability to localise the lesion, identify the metabolism of the tumour, and help in the staging and prognosis of PCL [17,18]. This was the main imaging technique which helped in unveiling the lymphoma mass arising from the pericardium and infiltrating into the atrium in our case. FDG PET-CT is very useful in detecting an intravascular large B cell lymphoma in the background of PUO or suspicion of malignancy, when no obvious abnormalities are detected on CT/MRI [19].

Diagnosis depends purely on histopathology and immunohistochemistry to identify, classify, and check the proliferative activity. Most of the NHL are B cell in origin, expressing CD20, CD79a, CD19, and other B-cell markers [10]. The prognosis of DLBCL, as per the literature, is poor especially when there is infiltration into the myocardium.

The main treatment aspects in PCL are chemotherapy, radiotherapy, and surgery depending on the situation of the tumour. Chemotherapy alone has shown remission in 61% of the cases and has overall shown to be highly effective [20]. DLBCL being the most common type, early initiation of chemotherapy tends to be effective due to its high invasiveness [13]. The present recommended regimen consists of R-CHOP or R-EPOCH (rituximab, etoposide, prednisone, vincristine, cyclophosphamide and doxorubicin). In our case, the tumour had involved the entire pericardium and ventricular myocardium with infiltration into the right atrium, superior mediastinum, and the great vessels.

The most common cause of death in PCL was heart failure, followed by sepsis, and progression of the lymphoma. The others which occur in a smaller percentage are arrhythmia, embolism, cerebrovascular accident, and sudden cardiac death [13].

## 4. Conclusions

In patients presenting with clinical features of fatigue, PUO, raised ESR/CRP, and anaemia of chronic disease it is critical that physicians search for the disease focus in obscure locations such as the cardia and differentiate PCL from relatively common cardiac tumours such as atrial myxoma. PCL, though extremely rare, merits consideration in patients with disproportionate dyspnoea, PUO, and symptoms of thoracic outlet obstruction. The difficulty in diagnosing the lesion, owing to the location and vague presenting symptoms and signs, often leads to delayed diagnosis and poor prognosis. PET-CT is an invaluable tool in patients with PUO, especially caused by neoplasms as it helps in localizing the target lesion, aiding in selecting the appropriate intervention for rapid tissue diagnosis.

## Data Availability

Data is unavailable due to privacy.
